# From Movements to Metrics: Evaluating Explainable AI Methods in Skeleton-Based Human Activity Recognition

**DOI:** 10.3390/s24061940

**Published:** 2024-03-18

**Authors:** Kimji N. Pellano, Inga Strümke, Espen A. F. Ihlen

**Affiliations:** 1 Department of Neuromedicine and Movement Science, Faculty of Medicine and Health Sciences, Norwegian University of Science and Technology, 7034 Trondheim, Norway; espen.ihlen@ntnu.no; 2 Department of Computer Science, Faculty of Information Technology and Electrical Engineering, Norwegian University of Science and Technology, 7034 Trondheim, Norway; inga.strumke@ntnu.no

**Keywords:** explainable AI, CAM, Grad-CAM, skeleton data, human activity recognition

## Abstract

The advancement of deep learning in human activity recognition (HAR) using 3D skeleton data is critical for applications in healthcare, security, sports, and human–computer interaction. This paper tackles a well-known gap in the field, which is the lack of testing in the applicability and reliability of XAI evaluation metrics in the skeleton-based HAR domain. We have tested established XAI metrics, namely faithfulness and stability on Class Activation Mapping (CAM) and Gradient-weighted Class Activation Mapping (Grad-CAM) to address this problem. This study introduces a perturbation method that produces variations within the error tolerance of motion sensor tracking, ensuring the resultant skeletal data points remain within the plausible output range of human movement as captured by the tracking device. We used the NTU RGB+D 60 dataset and the EfficientGCN architecture for HAR model training and testing. The evaluation involved systematically perturbing the 3D skeleton data by applying controlled displacements at different magnitudes to assess the impact on XAI metric performance across multiple action classes. Our findings reveal that faithfulness may not consistently serve as a reliable metric across all classes for the EfficientGCN model, indicating its limited applicability in certain contexts. In contrast, stability proves to be a more robust metric, showing dependability across different perturbation magnitudes. Additionally, CAM and Grad-CAM yielded almost identical explanations, leading to closely similar metric outcomes. This suggests a need for the exploration of additional metrics and the application of more diverse XAI methods to broaden the understanding and effectiveness of XAI in skeleton-based HAR.

## 1. Introduction

Analyzing human movement through 3D skeleton data has promising nontrivial applications in high-stake sectors such as healthcare and rehabilitation [1], security and surveillance [2], sports and athletics [3], and human–computer interaction [4]. Because of this, integrating deep learning in skeleton data analysis requires an understanding of the model’s decision-making processes. One particular application is compliance with the EU’s proposed AI Act, which emphasizes that transparency and human oversight should be embedded in high-risk applications such as AI-assisted medical diagnostics [5]. A basic form of deep learning technique applied to human movement analysis using skeleton data is human activity recognition (HAR). State-of-the-art HAR models are continually being developed and improved. It started with the introduction of Spatial–Temporal Graph Convolutional Network (ST-GCN) architectures in 2018 [6], as they were the first to use graph convolution for HAR. ST-GCN then became the baseline for dozens of emerging skeleton-based HAR models that seek to improve this original implementation.

Recent advancements in HAR model architectures have been significant, but strides in their explainability remain limited. Class Activation Mapping (CAM) was used in EfficientGCN [7] and ST-GCN [8] to highlight the body points significant for specific actions. In [9], Gradient-weighted Class Activation Mapping (Grad-CAM) was implemented in ST-GCN. There is a growing trend towards using explainable AI (XAI) methods, extending from CNNs to ST-GCNs, yet XAI metrics to assess their reliability in this domain have yet to be tested. There is also a lack of comparative analysis between these methods, which leaves a gap in understanding their relative performance in HAR applications. Additionally, research is limited regarding metrics that assess XAI methods in the context of data perturbation.

While the paper in [9] evaluated the faithfulness and contrastivity of Grad-CAM, it did not offer insights into its performance relative to other XAI methods. Moreover, their choice of using masking/occlusion to check for changes in prediction output raises concerns. Masking can potentially distort the standard human skeletal structure that GCN-based models are trained on. Movements of the human body are governed by biomechanical principles, and perturbations that do not respect these principles can potentially result in a misleading understanding of the model’s faithfulness. Recognizing the growing relevance of skeleton-based HAR models in critical areas, our paper aims to test established metrics that assess their corresponding feature attribution techniques. Additionally, this study pioneers the evaluation of explainability metrics within the context of perturbations that fall within the error tolerance of depth-sensing technology, such as that of the Kinect device, effectively simulating realistic variations in skeletal data. In [10,11], the perturbation of skeleton data was employed, which they claim maintained normal human kinematics during perturbation. However, the objective was for an adversarial attack; thus, the perturbed skeleton joints were neither controlled nor deliberately targeted. In essence, our work also addresses the gap in evaluation metrics by leveraging targeted perturbations that align with the Kinect device’s error tolerance, providing a practical approach to simulate realistic skeletal data variations.

Alongside the pursuit of improved explainability, assessing the stability of model decisions and their explanations is important. As human skeletal data can exhibit subtle variances due to minor changes in posture, movement, or data-capturing techniques, the decisions from the model and the explanations from the XAI methods should remain consistent and trustworthy. That is, dramatic shifts in explanations due to minor input changes cast doubt on model reliability. Moreover, the imprecise estimation of joint center positions in 3D skeletal data analysis underscores the need to evaluate decision and explanation robustness using perturbations that remain within the realistic operational bounds of the capturing device’s tracking capabilities. To address this, we draw from metrics established for other data types. In this work, we focus on the two primary metrics highlighted in [12]: faithfulness, which gauges how closely an explanation mirrors the model’s internal logic, and stability, which pertains to the consistency of a model’s explanations across similar inputs.

This paper’s key contributions are as follows:

- Testing established metrics and assessing their applicability for evaluating XAI methods in skeleton-based HAR.
- Introducing a controlled approach to perturb 3D skeleton data for XAI evaluation that accommodates realistic variations within the inherent inaccuracies of the tracking device.
- Assessing the impact of perturbation magnitude variations on metrics.
- Comparing the performance of CAM, Grad-CAM, and a random feature attribution method for HAR.

## 2. Materials

To provide the framework for this research, the dataset used, the neural network architecture trained and tested, and the XAI metrics implemented are briefly summarized below.

### 2.1. NTU RGB+D 60 Dataset and EfficientGCN

The NTU RGB+D 60 dataset [13] contains 60 action classes with over 56 thousand 3D skeleton data, each composed of sequential frames captured from 40 different subjects using the Kinect v2 camera with depth sensor. For evaluation purposes, the dataset is further divided into cross-subject and cross-view subgroups—the former is composed of different human subjects performing the same actions, while the latter uses different camera angle views.

The EfficientGCN architecture [14] is a result of extending the concept of EfficientNet [15] for CNNs to ST-GCNs to reduce the computing resource demand for HAR. There are a total of 24 different EfficientGCN network configurations with different scaling coefficients that the user can choose and test. In this paper, we use the B4 configuration, which has achieved the highest accuracy at 92.1% on the cross-subject dataset and 96.1% on cross-view, compared with the 81.5% and 88.3% of the baseline ST-GCN, respectively.

### 2.2. Evaluation Metrics

#### 2.2.1. Faithfulness

In XAI, fidelity or faithfulness, as described in [16,17,18], measures how well an explanation shows what truly influences a model’s decisions, focusing on the importance of different features. This concept checks if the explanation accurately matches the actual effect of these features on the model’s predictions, providing a crucial way to judge if explanations are trustworthy. Both the impact of important and unimportant features, as identified by the XAI method, can be quantitatively assessed for their accuracy in reflecting the model’s decision-making process, with the mathematical framework for this assessment provided in [12]. The Prediction Gap on Important feature perturbation (PGI) measures how much prediction changes when top-*k* features are perturbed. Conversely, the Prediction Gap on Unimportant feature perturbation (PGU) measures the change in prediction when unimportant features are perturbed. Let *X* represent the original input data with its associated explanation $e_{X}$ and f(·) represent the output probability. Then, $X^{\prime }$ signifies the perturbed variant of *X* and $e_{X}^{\prime }$ the revised explanation after perturbation.

(1) $$PGI\left(X,\;f,\;e_{X},\;k\right)=E_{X^{\prime }∼\mathrm{perturb}\left(X,e_{X},\mathrm{top}-k\right)}[|f\left(X\right)-f\left(X^{\prime }\right)\left|\right]$$

(2) $$PGU\left(X,\;f,\;e_{X},\;k\right)=E_{X^{\prime }∼\mathrm{perturb}\left(X,e_{X},\mathrm{non}\;\mathrm{top}-k\right)}[|f\left(X\right)-f\left(X^{\prime }\right)\left|\right]$$

#### 2.2.2. Stability

The concept of stability, also known as robustness, refers to the maximum amount of change in explanation (i.e., attribution scores) when the input data are slightly perturbed, as defined in [19,20]. The idea is that when original data are slightly perturbed, the resulting explanation should not drastically change, ensuring the model’s interpretations are stable and reliable across minor variations.. There are three submetrics that can be calculated, as enumerated in [12].

Relative Input Stability (RIS) measures the maximum change in attribution scores with respect to a corresponding perturbation in the input. Given that EfficientGCN has multiple input branches, it is essential to compute the RIS for each branch namely joint, velocity, and bone. From hereon, they are referred to as RISj, RISv, and RISb, respectively.

(3) $$\begin{array}{cc} \mathrm{RIS}\left(X,X^{\prime },e_{X},e_{X^{\prime }}\right) & =\max_{X^{\prime }}\frac{{∥\frac{\left(e_{X}-e_{X^{\prime }}\right)}{e_{X}}∥}_{p}}{\max\left({∥\frac{\left(X-X^{\prime }\right)}{X}∥}_{p},\epsilon_{\min}\right)},\\ \; & \;\forall X^{\prime }\;s.t.\;X^{\prime }\in N_{X};f\left(X\right)=f\left(X^{\prime }\right) \end{array}$$

Relative Output Stability (ROS) measures the maximum ratio of how much the explanation changes to how much the model’s prediction probability changes due to small perturbations in the input data.

(4) $$\begin{array}{cc} \mathrm{ROS}\left(X,X^{\prime },e_{X},e_{X^{\prime }}\right) & =\max_{X^{\prime }}\frac{{∥\frac{\left(e_{X}-e_{X^{\prime }}\right)}{e_{X}}∥}_{p}}{\max\left({∥\frac{\left(f\left(X\right)-f\left(X^{\prime }\right)\right)}{f(X)}∥}_{p},\epsilon_{\min}\right)}\\ \; & \;\forall X^{\prime }\;s.t.\;X^{\prime }\in N_{X};f\left(X\right)=f\left(X^{\prime }\right) \end{array}$$

Relative Representation Stability (RRS) measures the maximum change in a model’s explanations relative to changes in the model’s internal representations brought about by small input perturbations. In this context, the internal representation denoted as $L_{X}$ typically refers to an intermediate layer’s output in a neural network, capturing the model’s understanding of the input data. In our experiment, we extract and use the logits from the layer preceding the softmax function for our computations.

(5) $$\begin{array}{cc} \mathrm{RRS}\left(X,X^{\prime },e_{X},e_{X^{\prime }}\right) & =\max_{X^{\prime }}\frac{{∥\frac{\left(e_{X}-e_{X^{\prime }}\right)}{e_{X}}∥}_{p}}{\max\left({∥\frac{\left(L_{X}-L_{X^{\prime }}\right)}{L_{X}}∥}_{p},\epsilon_{\min}\right)}\\ \; & \;\forall X^{\prime }\;s.t.\;X^{\prime }\in N_{X};f\left(X\right)=f\left(X^{\prime }\right) \end{array}$$

## 3. Methods

### 3.1. Skeleton Data Perturbation

In 3D space, skeleton joints can be perturbed using spherical coordinates by generating random angles θ and ϕ for perturbation direction, sourced from a Gaussian distribution. The magnitude of this perturbation is controlled by radius *r*. In a standard XAI metric test, we recommend adhering to the principle that $X^{\prime }$ should be within the neighborhood of *X* to ensure that inputs remain representative of human kinematics and avoid skewing model predictions. This means constraining the magnitude of *r*, which in our pipeline is initially set to 2.5 cm. When it comes to body point tracking, the Kinect v2’s tracking error ranges from 1 to 7 cm compared with the gold-standard Vicon system [21], so a 2.5 cm perturbation ensures the perturbed data stay within Kinect’s typical accuracy tolerance. However, we also tested the metrics with increasing *r* (in cm: 2.5, 5, 10, 20, 40, and 80). While this contradicts our initial recommendation, varying the perturbation magnitude would allow us to (a) test the hypothesis that a small perturbation should result in meaningful changes in the prediction, which should be reflected in faithfulness results, and (b) observe its effects on the explanations, which should be reflected in the stability results.

**Figure 1:**
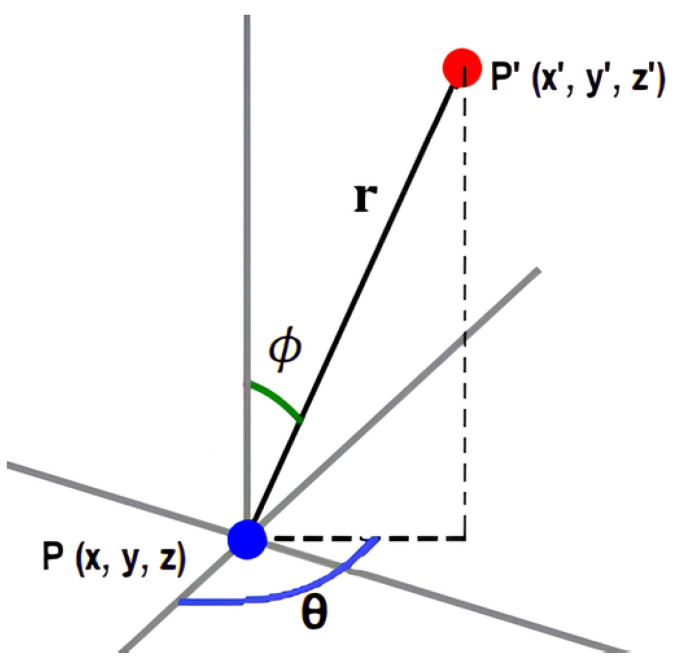
Illustration of perturbing a point P(x, y, z) in 3D space to a new position P′(x′, y′, z′) using spherical coordinates. The perturbation magnitude is represented by *r*, with azimuthal angle θ and polar angle ϕ.

The point P′(x′, y′, z′) in Figure 1 can be calculated using the equations below, which are used to convert a point from spherical to Cartesian (rectangular) coordinates. In these equations, *r* represents the distance from the two points, θ denotes the azimuthal angle, and ϕ is the polar angle. A fixed random seed was used to generate reproducible angles θ and ϕ. The variables dx, dy, and dz are computed once, and each joint is given its own unique set of these values. When added to the original coordinates across all video frames, a mildly perturbed 3D point is produced. This method ensures that a particular joint undergoes the same random adjustment across all frames, rather than different perturbations in each frame.

$$\begin{array}{cccc} x^{\prime } & =x+dx,\; & dx & =r\sin(\phi )\cos(\theta )\\ y^{\prime } & =y+dy,\; & dy & =r\sin(\phi )\sin(\theta )\\ z^{\prime } & =z+dz,\; & dz & =r\cos(\phi ) \end{array}$$

### 3.2. Calculation and Evaluation of XAI Metrics

**Figure 2:**
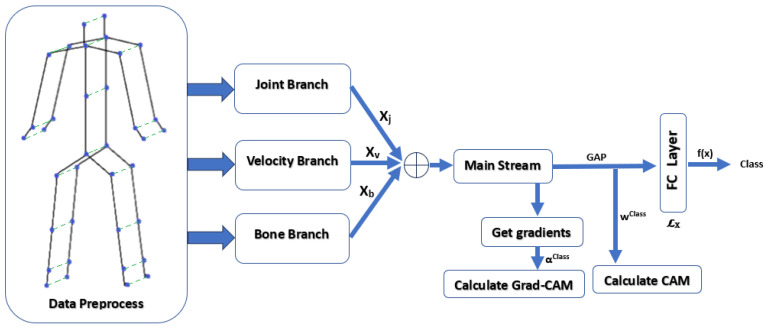
The EfficientGCN pipeline showing the variables for calculating faithfulness and stability. Perturbation is performed in the Data Preprocess stage.

**Figure 3:**
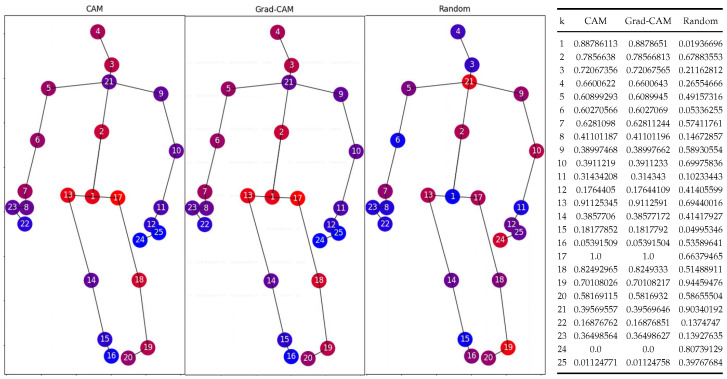
(**left**) CAM, Grad-CAM, and baseline random attributions for a data instance in ‘standing up’ (class 8), averaged for all frames and normalized. The color gradient denotes the score intensity: blue indicates 0, and progressing to red indicates a score of 1; (**right**) the numerical values of the attribution scores, with k denoting the body point number.

To calculate the metric values for a given action class, we employ the Area Under the Curve (AUC) to aggregate results into a single measure. Our process involves initializing variables and perturbing each data instance to n=50 times at set perturbation magnitudes. For each data instance, we input the original skeleton data into the model, capturing the explanation as detailed in Equations (6) and (7) for CAM and Grad-CAM, respectively. The model output and other necessary variables are also obtained as shown in the EfficientGCN pipeline [14] in Figure 2. To ensure metrics comparability across XAI methods, we normalize attribution scores between 0 and 1, and then rank features from highest to lowest based on the average score across all video frames. From the definition of CAM [22], *w* in Equation (6) are the weights after Global Average Pooling (GAP) for the specific output class, and $F^{n}$ denotes the *n*th feature map. Similarly, α in Equation (7) for Grad-CAM [23] is calculated by averaging the gradients. Figure 3 shows sample CAM and Grad-CAM explanations in comparison with the random baseline. More samples can be found in Appendix B.

(6) $$e_{X,\;\mathrm{CAM}}=\sum_{n}w_{n}^{class}F^{n}$$

(7) $$e_{X,\;\mathrm{Grad}-\mathrm{CAM}}=\sum_{n}\alpha_{n}^{class}F^{n}$$

Next, we systematically perturb the top-k body points (k = 1 to 25) *n* times, calculating the PGI as per Equation (1), and similarly perturb the remaining points to compute the PGU using Equation (2). Stability metrics are derived from the original and new explanations using Equations (3)–(5). Finally, we calculate the AUC for each metric across all k values for each data instance. The mean and standard deviation of these AUCs across all instances in a class provide the overall metric values.

Since the metrics are unitless, a random method serves as the benchmark for the least desirable outcome by randomly assigning feature attribution scores. Higher PGI values are optimal, indicating that altering important skeletal nodes has a marked impact on the prediction. Lower PGU values are better, suggesting that perturbing the identified unimportant skeletal nodes does not cause a significant change in the model’s output prediction. Lastly, a stability (RIS, RRS, and ROS) closer to zero is indicative of a model’s robustness, signifying that minor perturbations to the input data do not lead to significant changes in the explanation. In order to thoroughly assess the applicability and consistency of the XAI evaluation metrics, we test them on both the most accurately classified class (class 26—‘jump up’) and the class with the highest misclassification rate (class 11—‘writing’) in the NTU dataset. Of the 276 samples in the class 26 test set, only 1 sample was misclassified by the EfficientGCN-B4 model, while only 174 were correctly classified out of 272 samples in class 11. With 4 parallel Tesla V100 GPUs in our hardware setup, the total test time to generate one table in the Appendix A section was about 165 h.

To assess the generalizability of our findings further, we also extended our evaluation to include the metrics of Class 32 (representing the action ‘checking time on watch’) and Class 9 (representing the action ‘clapping’). For comparison, the model accurately predicted 253 out of 276 test instances for Class 32, and 221 out of 273 test instances for Class 9. Detailed graphical representations of these results, along with their corresponding numerical values, are presented in Appendix A Table A3 and Table A4, respectively.

## 4. Results

**Figure 4:**
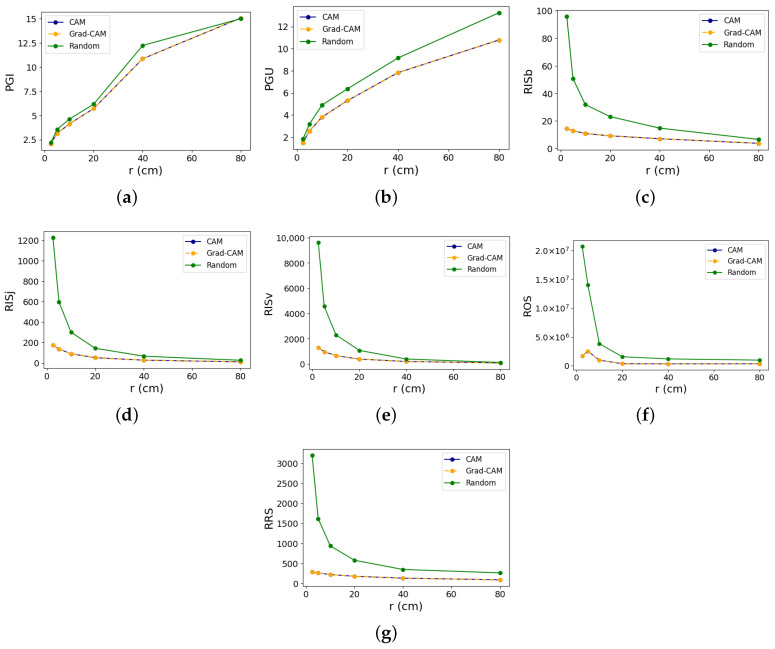
Evaluation metric outcomes for ‘Writing’ (Class 11, i.e., the weakest class), showing CAM (blue), Grad-CAM (orange), and the random (green) methods for (**a**) PGI, (**b**) PGU, (**c**) RISb, (**d**) RISj, (**e**) RISv, (**f**) ROS, and (**g**) RRS. The *y*-axis measures the metric values, while the *x*-axis shows the perturbation magnitude. CAM and Grad-CAM graphs overlap due to extremely similar metric outcomes.

**Figure 5:**
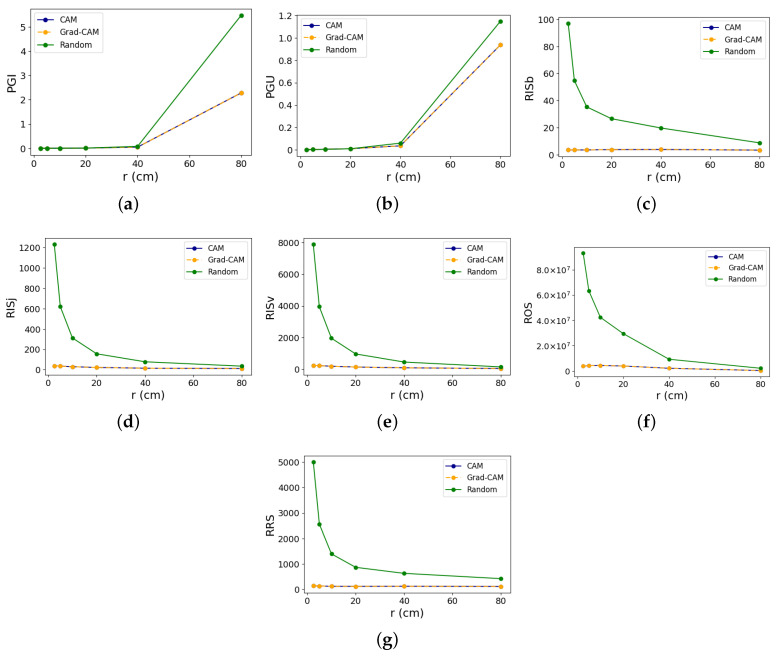
Evaluation metric outcomes for ‘Jump Up’ (Class 26, i.e., the strongest class), showing CAM (blue), Grad-CAM (orange), and the random (green) methods for (**a**) PGI, (**b**) PGU, (**c**) RISb, (**d**) RISj, (**e**) RISv, (**f**) ROS, and (**g**) RRS. The *y*-axis measures the metric values, while the *x*-axis shows the perturbation magnitude. CAM and Grad-CAM graphs overlap due to extremely similar metric outcomes.

To help us gauge the reliability of the XAI evaluation metrics, we tested them by slowly increasing the perturbation magnitude, as described in the Section 3. Figure 4 and Figure 5 show the line graphs for each metric test on class 11 and 26, respectively, comparing the different XAI methods. From hereon, we refer to class 26 as the strongest class, while class 11 is referred to as the weakest class. For the exact numerical values of the results with confidence intervals, please refer to Appendix A Table A1 and Table A2.

### 4.1. Faithfulness

**Figure A1:**
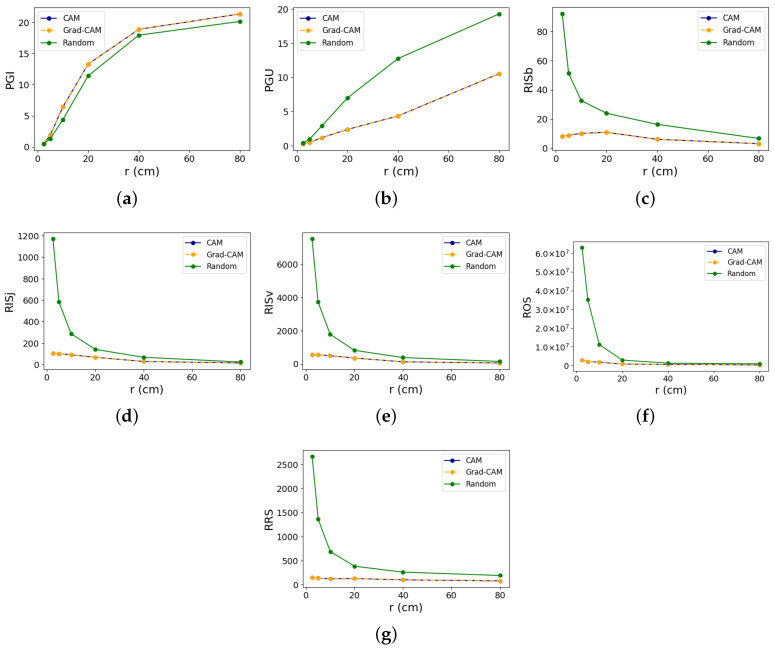
Evaluation metric outcomes for ‘checking time on watch’ (Class 32), showing CAM (blue), Grad-CAM (orange), and the random (green) methods for (**a**) PGI, (**b**) PGU, (**c**) RISb, (**d**) RISj, (**e**) RISv, (**f**) ROS, and (**g**) RRS. Despite increasing perturbation magnitudes, CAM and Grad-CAM exhibit only marginally better performance compared with the random method in terms of PGI. This suggests that the expected correlation between increasing the perturbation magnitude of important features and significant changes in prediction output may not consistently apply to this particular case. Conversely, for PGU, CAM and Grad-CAM demonstrate more effective identification of unimportant features compared with the random method. As perturbation magnitude increases, the random method results in a significantly larger discrepancy between the prediction probabilities of the original and perturbed data.

**Figure A2:**
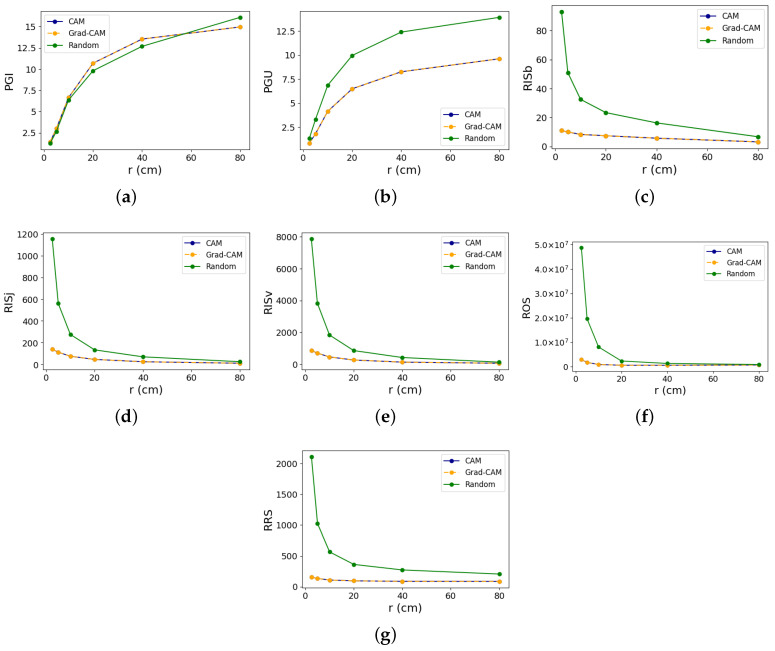
Evaluation metric outcomes for ‘clapping’ (Class 9), showing CAM (blue), Grad-CAM (orange), and the random (green) methods for (**a**) PGI, (**b**) PGU, (**c**) RISb, (**d**) RISj, (**e**) RISv, (**f**) ROS, and (**g**) RRS. Similar to class 32, the PGI results for class 9 show only a slight difference in performance between CAM and Grad-CAM versus the random method, even as perturbation magnitude increases. The PGU results still echo those in class 32, with CAM and Grad-CAM outperforming the random method in distinguishing unimportant features.

The identical PGI and PGU values for CAM and Grad-CAM mean both methods have the exact same ranking of features, although the numerical attribution scores are not exactly the same. Unexpectedly, the random method appears to outperform both in PGI in the weakest class, except at *r* = 80 cm. Conversely, looking at the results for the strongest class in Figure 5 suggests equal PGI performance among all methods up to r≤5 cm, beyond which the random method seems to surpass the others. In essence, in a class where the model has the best classification performance, the PGI test aligns with expected outcomes only at higher values of *r*, where data distortion is significant, which is no longer consistent with the rule that $X^{\prime }\in N_{X}$. Moreover, where model classification is the weakest, PGI results consistently give unexpected outcomes. In class 32 (results shown in Figure A1), which has a classification accuracy of 91.67%, both CAM and Grad-CAM outperform the random method in terms of PGI, albeit with a small margin. Conversely, in class 9 (results shown in Figure A2), with a classification accuracy of 80.95%, CAM and Grad-CAM also outperform the random method across all perturbation magnitudes, except at r=80 cm.

An analysis of the PGU results of the weakest class indicates that CAM and Grad-CAM outperform the random method, as expected. In the strongest class, however, conformity to expected outcomes occurs only when r≥40 cm, with random exhibiting higher values, while in lower perturbation magnitudes, the results seem to suggest that the three methods exhibit either comparable performance or that the random method has marginally higher PGU values. Therefore, PGU tests only meet expected outcomes primarily when there is weak model performance or when input perturbation is significant during strong model performance. For classes 32 and 9, PGU scores of both CAM and Grad-CAM surpass the random method, as anticipated, with increasing margins as perturbation magnitude increases.

These irregularities in both PGI and PGU suggest that the hypothesis that faithfulness is anchored on—minor perturbations causing meaningful prediction shifts—is not upheld in all action classes of the NTU dataset for the EfficientGCN model. Using output predictions for gauging explanation fidelity proves unreliable in this context.

### 4.2. Stability

Stability assessments for CAM and Grad-CAM yield nearly identical values, diverging only in less significant decimal places. This implies that despite both methods giving different raw scores, they tend to converge upon normalization. Stability test results, contrary to faithfulness, demonstrate robustness against increased perturbation, consistently indicating the superiority of CAM and Grad-CAM over random in the four classes tested. Thus, stability testing affirms that input perturbations do not drastically alter explanations compared with the random baseline.

It can be observed in Figure 4f and Figure 5f that ROS results register very high numerical values, with the *y*-axis scaled to $1\times 10^{7}$. We inspected the individual terms in each of the ROS results and found that the cause for such high numbers is the extremely small denominator terms (typically less than 1). Since the denominator term of ROS is the difference between the original and perturbed predictions, it means that the change in the model’s output probability is very small, even when the perturbation magnitude is large. A small denominator, reflecting little changes in output probabilities even with substantial perturbations, corroborates the inefficacy of the PGI and PGU tests in our context. These tests, which are reliant on shifts in prediction probabilities, fail to yield meaningful results in response to input perturbations, further supporting our hypothesis regarding the model’s behavior under examination.

## 5. Discussion and Conclusions

Our research contributes to the understanding of explainable AI in the context of skeleton-based HAR, advancing the state-of-the-art by testing known metrics in this emerging domain and introducing a perturbation technique informed by the practical constraints of skeletal tracking technology. A key finding from our experiments is that faithfulness—a widely recognized XAI metric—may falter in certain models, such as the EfficientGCN-B4 tested in this study. This finding serves as a caution to XAI practitioners when using an XAI metric that measures the reliability of XAI methods indirectly through the change in the model’s prediction probability. In contrast, stability, which measures direct changes in explanations, emerged as a dependable metric. However, this leaves us with only a single metric, which offers a limited view of an XAI method’s efficacy, underscoring the need for developing or adapting additional testing approaches in this field. Our skeleton perturbation method, which simulates realistic variations within the operational tolerances of skeletal tracking technologies, offers a promising framework for validating upcoming XAI metrics.

This study also identifies other gaps in XAI for ST-GCN-based HAR, which is an opportunity for future research directions. The nearly identical explanations produced by Grad-CAM and CAM when applied to EfficientGCN highlight a need for more diverse XAI techniques, such as adaptations of model agnostic methods like LIME [24] and SHAP [25] for this specific domain. Additionally, comparative studies of XAI metrics across different existing HAR models can also be explored, which could be valuable as a guide for model selection where explainability is as important as accuracy.

Lastly, our comparative analysis between CAM and Grad-CAM revealing negligible stability differences suggests that neither method is superior; they are essentially equivalent. Yet, CAM’s use of static model weights obtained post training means it demands less computational load compared with Grad-CAM, which needs gradient computation per data instance. This highlights CAM’s suitability for large-scale data analysis. This consideration is especially pertinent for applications where computational efficiency is vital alongside accuracy and reliability.

## Data Availability

The dataset used in the paper is openly accessible and does not require any authorization or permission: NTU-RGB+D 60 (https://rose1.ntu.edu.sg/dataset/actionRecognition/ accessed on 24 October 2022).

## Appendix A

**Table A1:** Class 11 tabular data: ↑ indicates that higher values are better, while ↓ indicates that lower values are optimal.

Method	r (cm)	PGI ↑	PGU ↓	RISj	RISv	RISb	ROS	RRS
CAM	2.5	2.080 ± 0.394	1.507 ± 0.268	174.133 ± 16.946	1288.370 ± 138.912	14.424 ± 1.202	1,702,553.834 ± 1,116,223.129	285.874 ± 46.673
	5	3.142 ± 0.514	2.547 ± 0.394	137.460 ± 11.303	968.090 ± 86.855	12.924 ± 0.964	2,530,301.100 ± 2,226,508.985	264.838 ± 33.044
	10	4.109 ± 0.569	3.807 ± 0.489	91.225 ± 6.774	654.886 ± 53.711	10.877 ± 0.741	971,050.496 ± 676,047.094	222.611 ± 20.795
	20	5.722 ± 0.616	5.294 ± 0.541	52.854 ± 3.473	380.917 ± 30.189	9.210 ± 0.556	355,153.157 ± 272,318.174	180.486 ± 11.691
	40	10.859 ± 0.811	7.826 ± 0.549	28.373 ± 2.139	190.976 ± 18.548	7.112 ± 0.416	334,731.175 ± 221,923.029	132.782 ± 8.488
	80	15.059 ± 1.023	10.787 ± 0.679	12.199 ± 1.408	76.734 ± 10.911	3.764 ± 0.330	340,629.702 ± 291,218.222	92.569 ± 8.165
Grad-CAM	2.5	2.080 ± 0.394	1.507 ± 0.268	174.133 ± 16.946	1288.371 ± 138.912	14.424 ± 1.202	1,702,554.201 ± 1,116,223.411	285.874 ± 46.673
	5	3.142 ± 0.514	2.547 ± 0.394	137.460 ± 11.303	968.090 ± 86.855	12.924 ± 0.964	2,530,301.513 ± 2,226,509.277	264.838 ± 33.044
	10	4.109 ± 0.569	3.807 ± 0.489	91.225 ± 6.774	654.886 ± 53.711	10.877 ± 0.741	971,050.337 ± 676,047.046	222.611 ± 20.795
	20	5.722 ± 0.616	5.294 ± 0.541	52.854 ± 3.473	380.917 ± 30.189	9.210 ± 0.556	355,153.184 ± 272,318.220	180.486 ± 11.691
	40	10.859 ± 0.811	7.826 ± 0.549	28.373 ± 2.139	190.976 ± 18.548	7.112 ± 0.416	334,731.302 ± 221,923.109	132.782 ± 8.488
	80	15.059 ± 1.023	10.787 ± 0.679	12.199 ± 1.408	76.734 ± 10.911	3.764 ± 0.330	340,629.723 ± 291,218.252	92.569 ± 8.165
Random	2.5	2.190 ± 0.410	1.831 ± 0.306	1228.105 ± 43.441	9627.812 ± 502.591	95.762 ± 3.630	20,728,354.210 ± 14,139,843.294	3205.744 ± 333.826
	5	3.559 ± 0.577	3.165 ± 0.445	598.011 ± 21.123	4555.435 ± 238.309	50.612 ± 1.843	13,986,453.073 ± 9,801,817.526	1617.393 ± 160.022
	10	4.613 ± 0.664	4.905 ± 0.542	300.288 ± 11.303	2300.006 ± 112.759	31.848 ± 1.120	3,835,957.191 ± 2,481,844.039	940.518 ± 87.484
	20	6.173 ± 0.643	6.357 ± 0.596	144.791 ± 5.982	1069.703 ± 56.382	23.135 ± 0.976	1,563,644.666 ± 1,072,148.002	580.579 ± 46.894
	40	12.232 ± 0.820	9.155 ± 0.654	67.294 ± 3.949	386.528 ± 35.895	14.813 ± 0.768	1,187,301.059 ± 987,974.000	350.415 ± 26.835
	80	14.989 ± 1.008	13.253 ± 0.852	27.610 ± 2.828	115.425 ± 19.128	6.621 ± 0.418	978,432.004 ± 1,211,946.037	266.365 ± 27.954

**Table A2:** Class 26 tabular data: ↑ indicates that higher values are better, while ↓ indicates that lower values are optimal.

Method	r (cm)	PGI ↑	PGU ↓	RISj	RISv	RISb	ROS	RRS
CAM	2.5	0.002 ± 0.001	0.002 ± 0.001	38.783 ± 1.685	241.329 ± 10.033	3.504 ± 0.112	3,793,568.715 ± 599,734.044	137.439 ± 4.602
	5	0.003 ± 0.001	0.003 ± 0.001	35.563 ± 1.469	226.272 ± 8.821	3.447 ± 0.103	4,244,155.156 ± 614,303.198	130.751 ± 3.800
	10	0.005 ± 0.002	0.006 ± 0.003	29.652 ± 1.165	191.308 ± 7.224	3.452 ± 0.110	4,307,133.768 ± 667,017.130	116.767 ± 3.276
	20	0.010 ± 0.005	0.010 ± 0.004	22.343 ± 0.782	140.125 ± 5.027	3.698 ± 0.108	3,926,629.242 ± 573,923.634	114.482 ± 3.087
	40	0.051 ± 0.040	0.036 ± 0.014	15.016 ± 0.481	91.628 ± 3.543	3.793 ± 0.111	2,119,077.311 ± 355,383.073	119.608 ± 2.883
	80	2.281 ± 0.299	0.938 ± 0.140	11.679 ± 0.347	45.246 ± 2.827	3.324 ± 0.090	339,790.687 ± 75,217.805	110.699 ± 2.141
Grad-CAM	2.5	0.002 ± 0.001	0.002 ± 0.001	38.783 ± 1.685	241.329 ± 10.033	3.504 ± 0.112	3,793,569.048 ± 599,734.059	137.439 ± 4.602
	5	0.003 ± 0.001	0.003 ± 0.001	35.563 ± 1.469	226.272 ± 8.821	3.447 ± 0.103	4,244,156.522 ± 614,303.356	130.751 ± 3.800
	10	0.005 ± 0.002	0.006 ± 0.003	29.652 ± 1.165	191.308 ± 7.224	3.452 ± 0.110	4,307,133.975 ± 667,017.145	116.767 ± 3.276
	20	0.010 ± 0.005	0.010 ± 0.004	22.343 ± 0.782	140.125 ± 5.027	3.698 ± 0.108	3,926,629.059 ± 573,923.586	114.482 ± 3.087
	40	0.051 ± 0.040	0.036 ± 0.014	15.016 ± 0.481	91.628 ± 3.543	3.793 ± 0.111	2,119,077.265 ± 355,383.079	119.608 ± 2.883
	80	2.281 ± 0.299	0.938 ± 0.140	11.679 ± 0.347	45.246 ± 2.827	3.324 ± 0.090	339,790.661 ± 75,217.791	110.699 ± 2.141
Random	2.5	0.002 ± 0.001	0.002 ± 0.001	1233.418 ± 22.376	7891.926 ± 206.386	97.067 ± 1.616	93,264,563.501 ± 12,716,437.799	5008.974 ± 133.687
	5	0.003 ± 0.002	0.003 ± 0.001	619.974 ± 10.867	3948.918 ± 101.998	54.711 ± 0.727	63,286,627.152 ± 9,322,027.632	2557.063 ± 64.789
	10	0.006 ± 0.002	0.005 ± 0.002	312.310 ± 5.332	1958.503 ± 50.638	35.273 ± 0.333	42,392,862.818 ± 6,446,239.434	1393.590 ± 32.140
	20	0.012 ± 0.005	0.010 ± 0.004	156.615 ± 2.673	966.698 ± 25.445	26.557 ± 0.193	29,527,933.778 ± 4,113,487.016	864.778 ± 18.918
	40	0.080 ± 0.055	0.060 ± 0.027	77.913 ± 1.346	454.485 ± 13.903	19.684 ± 0.241	9,244,726.238 ± 1,352,133.114	627.195 ± 13.200
	80	5.474 ± 0.334	1.149 ± 0.176	35.110 ± 0.858	149.132 ± 7.404	8.684 ± 0.145	2,129,144.307 ± 407,137.423	419.232 ± 8.483

**Table A3:** Class 32 tabular data: ↑ indicates that higher values are better, while ↓ indicates that lower values are optimal.

Method	r (cm)	PGI ↑	PGU ↓	RISj	RISv	RISb	ROS	RRS
CAM	2.5	0.530 ± 0.172	0.275 ± 0.113	104.752 ± 12.666	567.154 ± 63.481	7.991 ± 1.036	2,916,268.239 ± 1,006,355.218	150.627 ± 22.877
	5	1.923 ± 0.466	0.469 ± 0.140	100.686 ± 9.080	547.544 ± 50.258	8.872 ± 0.831	2,155,297.199 ± 768,443.139	136.450 ± 18.700
	10	6.421 ± 0.793	1.169 ± 0.229	89.387 ± 6.276	492.629 ± 39.847	10.145 ± 0.645	1,787,187.499 ± 738,937.270	122.012 ± 13.391
	20	13.318 ± 0.765	2.366 ± 0.344	66.464 ± 5.372	352.318 ± 34.570	10.860 ± 0.728	836,212.856 ± 204,359.147	127.161 ± 9.837
	40	18.903 ± 0.679	4.338 ± 0.438	27.222 ± 2.617	124.864 ± 14.660	6.080 ± 0.462	628,225.894 ± 157,229.139	100.372 ± 6.547
	80	21.380 ± 0.637	10.536 ± 0.567	13.050 ± 1.496	53.424 ± 8.608	3.111 ± 0.243	314,147.370 ± 133,974.310	77.798 ± 5.265
Grad-CAM	2.5	0.530 ± 0.172	0.275 ± 0.113	104.752 ± 12.666	567.154 ± 63.481	7.991 ± 1.036	2,916,267.512 ± 1,006,355.183	150.627 ± 22.877
	5	1.923 ± 0.466	0.469 ± 0.140	100.686 ± 9.080	547.544 ± 50.258	8.872 ± 0.831	2,155,296.259 ± 768,443.051	136.450 ± 18.700
	10	6.421 ± 0.793	1.169 ± 0.229	89.387 ± 6.276	492.629 ± 39.847	10.145 ± 0.645	1,787,187.829 ± 738,937.896	122.012 ± 13.391
	20	13.318 ± 0.765	2.366 ± 0.344	66.464 ± 5.372	352.318 ± 34.570	10.860 ± 0.728	836,212.707 ± 204,359.110	127.161 ± 9.837
	40	18.903 ± 0.679	4.338 ± 0.438	27.222 ± 2.617	124.864 ± 14.660	6.080 ± 0.462	628,225.943 ± 157,229.158	100.372 ± 6.547
	80	21.380 ± 0.637	10.536 ± 0.567	13.050 ± 1.496	53.424 ± 8.608	3.111 ± 0.243	314,147.317 ± 133,974.305	77.798 ± 5.265
Random	2.5	0.516 ± 0.169	0.402 ± 0.140	1171.479 ± 26.522	7546.971 ± 238.181	92.114 ± 2.274	63,030,515.690 ± 17,269,283.238	2668.959 ± 142.801
	5	1.318 ± 0.333	0.928 ± 0.225	580.844 ± 13.855	3727.449 ± 119.082	51.361 ± 1.150	35,297,240.144 ± 10,033,888.566	1360.739 ± 71.238
	10	4.401 ± 0.625	2.923 ± 0.430	286.405 ± 6.934	1781.205 ± 57.356	32.552 ± 0.640	11,237,060.760 ± 3,678,599.967	688.543 ± 34.206
	20	11.434 ± 0.744	6.970 ± 0.681	139.891 ± 3.732	819.171 ± 35.888	23.906 ± 0.509	2,949,464.762 ± 1,189,151.206	383.922 ± 18.093
	40	17.962 ± 0.631	12.742 ± 0.789	66.049 ± 2.985	381.788 ± 25.718	16.294 ± 0.718	1,293,045.700 ± 455,084.066	260.787 ± 17.520
	80	20.176 ± 0.610	19.286 ± 0.592	22.288 ± 2.462	147.523 ± 15.526	6.688 ± 0.579	978,000.924 ± 471,660.208	191.267 ± 22.531

**Table A4:** Class 9 tabular data: ↑ indicates that higher values are better, while ↓ indicates that lower values are optimal.

Method	r (cm)	PGI ↑	PGU ↓	RISj	RISv	RISb	ROS	RRS
CAM	2.5	1.446 ± 0.299	0.834 ± 0.156	139.642 ± 9.088	859.375 ± 59.025	10.975 ± 0.749	2,813,109.184 ± 1,254,700.809	152.976 ± 8.557
	5	2.985 ± 0.499	1.811 ± 0.280	112.662 ± 6.406	693.715 ± 42.527	9.969 ± 0.545	1,667,239.107 ± 838,594.507	133.983 ± 6.921
	10	6.648 ± 0.727	4.144 ± 0.451	75.351 ± 4.038	453.420 ± 27.108	8.299 ± 0.409	825,855.283 ± 303,557.804	107.961 ± 5.679
	20	10.697 ± 0.786	6.490 ± 0.494	46.355 ± 2.759	260.949 ± 18.162	7.391 ± 0.375	518,198.588 ± 209,846.956	94.920 ± 5.303
	40	13.535 ± 0.703	8.259 ± 0.508	24.799 ± 1.502	127.559 ± 9.371	5.708 ± 0.299	516,925.509 ± 228,941.576	86.767 ± 4.868
	80	14.963 ± 0.661	9.610 ± 0.488	11.064 ± 1.075	57.528 ± 6.981	3.150 ± 0.150	611,673.611 ± 310,288.379	86.007 ± 4.309
Grad-CAM	2.5	1.446 ± 0.299	0.834 ± 0.156	139.642 ± 9.088	859.375 ± 59.025	10.975 ± 0.749	2,813,108.561 ± 1,254,700.783	152.976 ± 8.557
	5	2.985 ± 0.499	1.811 ± 0.280	112.662 ± 6.406	693.715 ± 42.527	9.969 ± 0.545	1,667,238.830 ± 838,594.559	133.983 ± 6.921
	10	6.648 ± 0.727	4.144 ± 0.451	75.351 ± 4.038	453.420 ± 27.108	8.299 ± 0.409	825,854.822 ± 303,557.678	107.961 ± 5.679
	20	10.697 ± 0.786	6.490 ± 0.494	46.355 ± 2.759	260.949 ± 18.162	7.391 ± 0.375	518,198.555 ± 209,846.916	94.920 ± 5.303
	40	13.535 ± 0.703	8.259 ± 0.508	24.799 ± 1.502	127.559 ± 9.371	5.708 ± 0.299	516,925.478 ± 228,941.539	86.767 ± 4.868
	80	14.963 ± 0.661	9.610 ± 0.488	11.064 ± 1.075	57.528 ± 6.981	3.150 ± 0.150	611,673.499 ± 310,288.287	86.007 ± 4.309
Random	2.5	1.279 ± 0.265	1.340 ± 0.251	1154.662 ± 32.520	7879.336 ± 286.308	93.075 ± 2.396	48,749,279.991 ± 19,674,016.058	2113.066 ± 106.944
	5	2.638 ± 0.426	3.309 ± 0.493	559.807 ± 17.176	3827.702 ± 145.161	50.903 ± 1.297	19,522,191.192 ± 8,735,689.839	1025.301 ± 50.038
	10	6.366 ± 0.634	6.861 ± 0.695	275.334 ± 9.421	1830.920 ± 78.622	32.519 ± 1.405	7,999,255.135 ± 3,838,244.128	565.638 ± 31.075
	20	9.807 ± 0.722	9.956 ± 0.719	134.102 ± 5.852	853.467 ± 42.655	23.369 ± 1.122	2,254,755.568 ± 1,106,476.034	362.369 ± 26.496
	40	12.680 ± 0.703	12.389 ± 0.691	69.849 ± 3.386	418.035 ± 24.775	16.281 ± 0.575	1,252,448.556 ± 804,210.707	272.395 ± 19.120
	80	16.093 ± 0.710	13.919 ± 0.640	25.073 ± 1.905	124.852 ± 12.648	6.706 ± 0.273	808,021.756 ± 771,280.581	205.893 ± 14.800

## Appendix B. Sample Data Instances and Their Corresponding CAM and Grad-CAM Scores (Normalized)

**Table A5:** Sample from class 9. k denotes body point number.

k	CAM	Grad-CAM
1	0.10499927	0.10499939
2	0.8457103	0.84571093
3	0.86337245	0.8633743
4	0.14498976	0.14498973
5	1.0	1.0
6	0.6217559	0.6217579
7	0.5937777	0.5937787
8	0.7676569	0.7676586
9	0.96693885	0.9669416
10	0.71324295	0.71324545
11	0.7423911	0.74239343
12	0.67857283	0.6785746
13	0.26158667	0.26158726
14	0.08999933	0.08999972
15	0.02405946	0.02405963
16	0.0	0.0
17	0.23806588	0.23806643
18	0.0483454	0.04834554
19	0.02031745	0.02031763
20	0.0140839	0.01408408
21	0.9171762	0.91717803
22	0.49637738	0.4963787
23	0.57773083	0.57773197
24	0.47685906	0.47686023
25	0.3491272	0.3491279

**Table A6:** Sample from class 11. k denotes body point number.

k	CAM	Grad-CAM
1	0.4192264	0.41922605
2	0.4485402	0.4485402
3	0.484537	0.484536
4	0.3869853	0.38698477
5	0.40052295	0.40052223
6	0.25756067	0.2575602
7	0.24608475	0.24608456
8	0.14658104	0.14658089
9	0.6106867	0.610686
10	0.7591647	0.75916386
11	1.0	1.0
12	0.69097036	0.69096994
13	0.33305895	0.3330585
14	0.19402055	0.19402048
15	0.0	0.0
16	0.08237603	0.08237588
17	0.37931275	0.3793123
18	0.18730809	0.18730754
19	0.04533133	0.04533128
20	0.07244555	0.07244562
21	0.47519997	0.47519904
22	0.11682785	0.11682767
23	0.23002054	0.23002037
24	0.72968245	0.7296809
25	0.8752001	0.87519866

**Table A7:** Sample from class 26. k denotes body point number.

k	CAM	Grad-CAM
1	0.6775505	0.67755353
2	0.8287168	0.8287187
3	1.0	1.0
4	0.7715871	0.77158725
5	0.7818266	0.78182703
6	0.72055995	0.72056246
7	0.77830803	0.7783087
8	0.3176544	0.31765595
9	0.72087973	0.72088116
10	0.76808375	0.76808643
11	0.6013477	0.6013488
12	0.34084404	0.3408448
13	0.7855472	0.78554976
14	0.6986992	0.6986986
15	0.44396198	0.44396254
16	0.35180664	0.35180822
17	0.7808226	0.78082335
18	0.839865	0.8398684
19	0.6782383	0.6782419
20	0.60077846	0.6007782
21	0.95954794	0.95955056
22	0.0	0.0
23	0.10886551	0.10886557
24	0.02142251	0.02142265
25	0.05266253	0.05266258

**Table A8:** Sample from class 32. k denotes body point number.

k	CAM	Grad-CAM
1	0.07923103	0.07923108
2	0.2850566	0.28505734
3	0.26772848	0.2677287
4	0.08914754	0.08914759
5	0.6860553	0.68605727
6	0.98223686	0.9822402
7	1.0	1.0
8	0.93831366	0.93831354
9	0.39556953	0.39556977
10	0.37355593	0.37355682
11	0.4707105	0.4707117
12	0.54854244	0.5485435
13	0.04235959	0.0423594
14	0.03665932	0.03665918
15	0.00418666	0.00418662
16	0.0063788	0.00637893
17	0.04925954	0.04925963
18	0.04581013	0.04581019
19	0.0	0.0
20	0.01638052	0.01638054
21	0.32532248	0.32532266
22	0.56411064	0.56411153
23	0.8676206	0.8676238
24	0.39054847	0.39054966
25	0.45242724	0.45242897

## References

[1] Nguyen T.N., Huynh H.H., Meunier J. (2016). Skeleton-based abnormal gait detection. Sensors.

[2] Liu C., Fu R., Li Y., Gao Y., Shi L., Li W. (2021). A self-attention augmented graph convolutional clustering networks for skeleton-based video anomaly behavior detection. Appl. Sci..

[3] Guo J., Liu H., Li X., Xu D., Zhang Y. (2021). An attention enhanced spatial–temporal graph convolutional LSTM network for action recognition in Karate. Appl. Sci..

[4] Usman M., Zhong J. (2022). Skeleton-based motion prediction: A survey. Front. Comput. Neurosci..

[5] Commission E. (2021). Proposal for a Regulation of the European Parliament and of the Council Laying Down Harmonised Rules on Artificial Intelligence (Artificial Intelligence Act) and Amending Certain Union legIslative Acts.

[6] Yan S., Xiong Y., Lin D. Spatial temporal graph convolutional networks for skeleton-based action recognition. Proceedings of the AAAI Conference on Artificial Intelligence.

[7] Song Y.F., Zhang Z., Shan C., Wang L. Stronger, faster and more explainable: A graph convolutional baseline for skeleton-based action recognition. Proceedings of the 28th ACM International Conference on Multimedia.

[8] Ghaleb E., Mertens A., Asteriadis S., Weiss G. (2021). Skeleton-based explainable bodily expressed emotion recognition through graph convolutional networks. Proceedings of the 2021 16th IEEE International Conference on Automatic Face and Gesture Recognition (FG 2021).

[9] Das P., Ortega A. (2022). Gradient-weighted class activation mapping for spatio temporal graph convolutional network. Proceedings of the ICASSP 2022—2022 IEEE International Conference on Acoustics, Speech and Signal Processing (ICASSP).

[10] Wang H., He F., Peng Z., Shao T., Yang Y.L., Zhou K., Hogg D. Understanding the robustness of skeleton-based action recognition under adversarial attack. Proceedings of the IEEE/CVF Conference on Computer Vision and Pattern Recognition.

[11] Liu J., Akhtar N., Mian A. (2020). Adversarial attack on skeleton-based human action recognition. IEEE Trans. Neural Netw. Learn. Syst..

[12] Agarwal C., Krishna S., Saxena E., Pawelczyk M., Johnson N., Puri I., Zitnik M., Lakkaraju H. (2022). Openxai: Towards a transparent evaluation of model explanations. Adv. Neural Inf. Process. Syst..

[13] Shahroudy A., Liu J., Ng T.T., Wang G. Ntu rgb+ d: A large scale dataset for 3d human activity analysis. Proceedings of the IEEE Conference on Computer Vision and Pattern Recognition.

[14] Song Y.F., Zhang Z., Shan C., Wang L. (2022). Constructing stronger and faster baselines for skeleton-based action recognition. IEEE Trans. Pattern Anal. Mach. Intell..

[15] Tan M., Le Q. Efficientnet: Rethinking model scaling for convolutional neural networks. Proceedings of the International Conference on Machine Learning.

[16] Alvarez Melis D., Jaakkola T. Towards robust interpretability with self-explaining neural networks. Proceedings of the Advances in Neural Information Processing Systems.

[17] Zhou J., Gandomi A.H., Chen F., Holzinger A. (2021). Evaluating the quality of machine learning explanations: A survey on methods and metrics. Electronics.

[18] Markus A.F., Kors J.A., Rijnbeek P.R. (2021). The role of explainability in creating trustworthy artificial intelligence for health care: A comprehensive survey of the terminology, design choices, and evaluation strategies. J. Biomed. Inform..

[19] Alvarez-Melis D., Jaakkola T.S. (2018). On the robustness of interpretability methods. arXiv.

[20] Agarwal C., Johnson N., Pawelczyk M., Krishna S., Saxena E., Zitnik M., Lakkaraju H. (2022). Rethinking stability for attribution-based explanations. arXiv.

[21] Otte K., Kayser B., Mansow-Model S., Verrel J., Paul F., Brandt A.U., Schmitz-Hübsch T. (2016). Accuracy and reliability of the kinect version 2 for clinical measurement of motor function. PLoS ONE.

[22] Zhou B., Khosla A., Lapedriza A., Oliva A., Torralba A. Learning deep features for discriminative localization. Proceedings of the IEEE Conference on Computer Vision and Pattern Recognition.

[23] Selvaraju R.R., Das A., Vedantam R., Cogswell M., Parikh D., Batra D. (2016). Grad-CAM: Why did you say that?. arXiv.

[24] Ribeiro M.T., Singh S., Guestrin C. “Why should I trust you?” Explaining the predictions of any classifier. Proceedings of the 22nd ACM SIGKDD International Conference on Knowledge Discovery and Data Mining.

[25] Lundberg S.M., Lee S.I. A unified approach to interpreting model predictions. Proceedings of the Advances in Neural Information Processing Systems.

